# An integrated strategy for improving contrast, durability, and portability of a Pocket Colposcope for cervical cancer screening and diagnosis

**DOI:** 10.1371/journal.pone.0192530

**Published:** 2018-02-09

**Authors:** Christopher T. Lam, Jenna Mueller, Betsy Asma, Mercy Asiedu, Marlee S. Krieger, Rhea Chitalia, Denali Dahl, Peyton Taylor, John W. Schmitt, Nimmi Ramanujam

**Affiliations:** 1 Department of Biomedical Engineering, Duke University, Durham, North Carolina, United States of America; 2 Duke Global Health Institute, Duke University, Durham, North Carolina, United States of America; 3 Center of Global Women’s Health Technologies, Duke University, Durham, North Carolina, United States of America; 4 Department of Obstetrics and Gynecology, Duke University Medical Center, Durham, North Carolina, United States of America; The Francis Crick Institute, UNITED KINGDOM

## Abstract

**Introduction:**

We have previously developed a portable Pocket Colposcope for cervical cancer screening in resource-limited settings. In this manuscript we report two different strategies (cross-polarization and an integrated reflector) to improve image contrast levels achieved with the Pocket Colposcope and evaluate the merits of each strategy compared to a standard-of-care digital colposcope. The desired outcomes included reduced specular reflection (glare), increased illumination beam pattern uniformity, and reduced electrical power budget. In addition, anti-fogging and waterproofing features were incorporated to prevent the Pocket Colposcope from fogging in the vaginal canal and to enable rapid disinfection by submersion in chemical agents.

**Methods:**

Cross-polarization (Generation 3 Pocket Colposcope) and a new reflector design (Generation 4 Pocket Colposcope) were used to reduce glare and improve contrast. The reflector design (including the angle and height of the reflector sidewalls) was optimized through ray-tracing simulations. Both systems were characterized with a series of bench tests to assess specular reflection, beam pattern uniformity, and image contrast. A pilot clinical study was conducted to compare the Generation 3 and 4 Pocket Colposcopes to a standard-of-care colposcope (Leisegang Optik 2). Specifically, paired images of cervices were collected from the standard-of-care colposcope and either the Generation 3 (n = 24 patients) or the Generation 4 (n = 32 patients) Pocket Colposcopes. The paired images were blinded by device, randomized, and sent to an expert physician who provided a diagnosis for each image. Corresponding pathology was obtained for all image pairs. The primary outcome measures were the level of agreement (%) and ***κ*** (*kappa)* statistic between the standard-of-care colposcope and each Pocket Colposcope (Generation 3 and Generation 4).

**Results:**

Both generations of Pocket Colposcope had significantly higher image contrast when compared to the standard-of-care colposcope. The addition of anti-fog and waterproofing features to the Generation 3 and 4 Pocket Colposcope did not impact image quality based on qualitative and quantitative metrics. The level of agreement between the Generation 3 Pocket Colposcope and the standard-of-care colposcope was 75.0% (*kappa* = 0.4000, p = 0.0028, n = 24). This closely matched the level of agreement between the Generation 4 Pocket Colposcope and the standard-of-care colposcope which was also 75.0% (*kappa* = 0.4941, p = 0.0024, n = 32).

**Conclusion:**

Our results indicate that the Generation 3 and 4 Pocket Colposcopes perform comparably to the standard-of-care colposcope, with the added benefit of being low-cost and waterproof, which is ideal for use in resource-limited settings. Additionally, the reflector significantly reduces the electrical requirements of the Generation 4 Pocket Colposcope enhancing portability without altering performance compared to the Generation 3 system.

## Introduction

The World Health Organization’s recommends a “see and treat” paradigm for cervical cancer prevention in low and middle-income countries. Current recommendations include human papilloma virus (HPV) testing (screening) and visual inspection with acetic acid (VIA) of HPV-positive women (diagnosis), followed by cryotherapy for women diagnosed with pre-cancerous lesions (treatment) [1]. If HPV testing is not available, visual inspection with acetic acid (VIA) is often used both as a screening and diagnosis tool [1]. However, VIA has several limitations including poor specificity, high inter-observer variability, and lack of image capture [2]. Several of these limitations could be addressed through the use of a low-cost portable colposcope as a screening or diagnostic tool at the primary care level.

Toward this end, two portable digital colposcopes have been commercialized: the Gynius AB’s Gynocular [3–5] and MobileODT’s EVA [6–8]. Both colposcopes have addressed some of these challenges by incorporating an on-board smartphone camera that can capture images at the point-of-care (see S1 Table for a comparison of system specifications and characteristics). While these colposcopes are portable, they rely on the cameras built into smartphones for digital image capture and can require a stand for stability. Consequently, these colposcopes have several limitations, which include a higher frequency of blurred images when compared to a standard-of-care colposcope and a higher prevalence of specular reflection [9,10].

**Fig 1:**
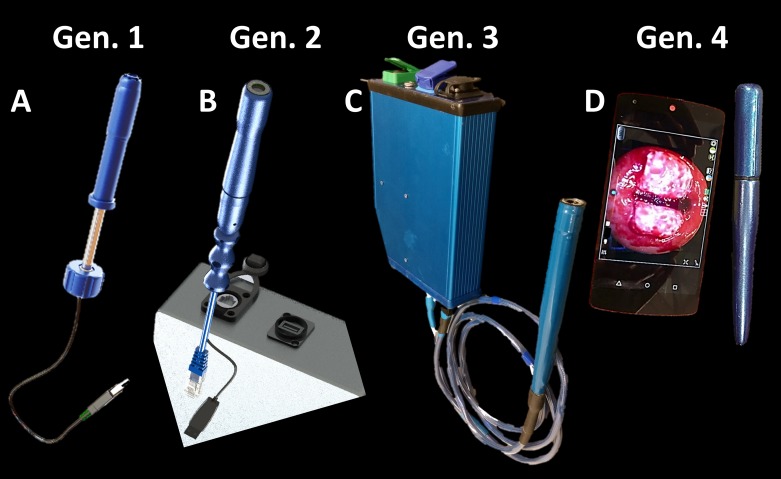
Evolution of the Pocket Colposcope design. (A) Generation 1 was our pilot proof-of-concept 5 megapixel color imager with only white LEDs; (B) the Generation 2 device added cross-polarization to reduce specular reflection, green LEDs allowing for enhanced vessel imaging, and an external voltage booster to boost LED intensity; (C) the Generation 3 device retains all features of Generation 2 system with the addition of a waterproofed case and a hydrophobic window to prevent fogging; (D) the Generation 4 device eliminated the need for cross-polarization and external voltage booster, through collimation and redirection of previously lost light back towards the cervix with a reflector tip.

To further address these limitations, we have developed a low-cost, transvaginal digital colposcope referred to as the Pocket Colposcope [11]. The Pocket Colposcope leverages light emitting diodes (LEDs), a consumer grade camera, and injection molded plastic lenses to greatly reduce the complexity, size, and cost compared to standard-of-care digital colposcopes. The Pocket Colposcope has gone through several design iterations, which are illustrated in Fig 1. The Generation 1 and 2 Pocket Colposcopes have previously been described in detail [11]. The Generation 1 Pocket Colposcope had reported issues including lens fogging, which required the use of anti-fog wipes, the need for ethylene oxide gas sterilization between each patient use due to lack of waterproofing, and issues of specular reflection that made it challenging to review images. To minimize specular reflection and maximize image contrast, the Generation 2 Pocket Colposcope employed the technique of cross-polarization [12–15]. Cross-polarization resulted in a ~75% signal reduction necessitating increased light delivery and requiring the use of an external control box with a voltage booster, specialized light source driving circuits, an external battery power supply, and a custom cable to plug the probe to into the external control box. First, a hydrophobic window was added to the Generation 3 Pocket Colposcope in order to prevent anti-fogging. Additionally, the device can withstand submersion in chemical agents including bleach or hydrogen peroxide for high-level disinfection between patient uses. The Generation 3 iteration retained the cross-polarizers and the voltage booster box of the Generation 2 Pocket Colposcope. However, providers disliked the stiffness of the required custom cable and the relative bulkiness of the external control box. The Generation 4 Pocket Colposcope was redesigned to maximize the efficiency of the on-board LEDs using a specialized reflector surface that does not require an external voltage booster. Due to the reduced voltage, the green LEDs that were incorporated into the Generation 2 and 3 Pocket Colposcopes were removed from the Generation 4 device.

In summary, this manuscript describes the computer-aided design optimization of the Pocket Colposcope in an effort to improve lighting efficiency and imaging contrast. Bench side verification testing of lighting efficiency and imaging contrast will quantify the level of improvement between our device generations. Lastly, we plan to demonstrate a comparable level of performance between these generations of the Pocket Colposcope to standard-of-care digital colposcope in a pilot clinical study.

## Materials and methods

### Crossed-polarization design for Generation 3

Glare or specular reflection can obfuscate proper diagnosis of precancerous cervical lesions, especially during VIA and colposcopy [16]. We elected to use cross-polarization [17,18] to reduce glare or specular reflection from cervical images during colposcopy. The Generation 2 and 3 Pocket Colposcopes have a linear glass polarizer placed (#43–783; Edmund Optics, Barrington NJ) in-line and parallel to the optical imaging axis. A second linear plastic film polarizer (#86–178; Edmund Optics, Barrington NJ) was placed over the illumination source (LEDs) at an orthogonal orientation to the imaging axis polarizer. An external voltage booster and constant current LED driving circuit were required to increase the illumination strength to compensate for the reduced illumination and imaging signals due to the set of polarizers [11].

### Reflector design and optimization for Generation 4

**Fig 2:**
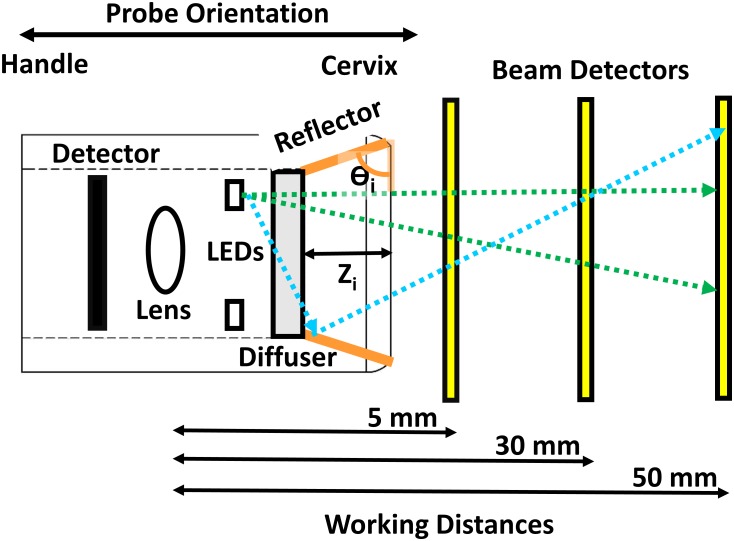
Schematic of reflector optimization ray tracing experiments. This figure shows the layout of our computer-aided optimization of the angle and height of the reflective surface with the probe tip facing the cervix to the right. The probe (left to right) contains the camera detector, lens, light emitting diode (LED) ring, LED diffuser, and reflector cone (orange). The geometric position and optical illumination properties of our LEDs were taken from manufacturer provided data files. A clear polycarbonate diffuser was modeled and placed over the concentric LED ring. Plate beam detectors were placed in the simulation at working distances from 5, 30, and 50 mm (yellow). These working distances are representative the range of highest, most commonly used, and lowest magnifications of our system. Three- dimensional models of each reflector design were placed in into the ray-tracing simulation. The reflector angles (Ɵ_i_) ranged from 0 to 75° degrees in 15° increments (orange). An outer probe diameter limit of 18 mm limited the range of reflector heights (Z_i_) from 0–4.82 mm. The effect of the reflector height alone was also investigated, where the reflector angle (Ɵ_i_) was fixed at 90° and the reflector heights (Z_i_) were varied from 0–4.82 mm.

A reflective surface was designed and optimized (height and angle) in order to provide consistent beam uniformity and increased optical power in the Generation 4 Pocket Colposcope. SolidWorks (Dassault Systemes; Waltham, MA) was used to create virtual polished aluminum reflectors with various heights ranging from 0 to 4.82 mm and angles of reflection ranging from 15° to 75° in 15° increments (Fig 2). These ranges of height and angle of the reflector were constrained by desired maximum outer diameter of the Generation 4 probe (20 mm). For each reflector design, simulations of light delivery were implemented using Zemax Optic Studio (Zemax LLC; Kirkland, WA). Manufacturer provided LED beam patterns and ray databases were used in our simulations and matched the exact make and model used by our prototypes. Our simulation parameters were set using 499,000 rays per LED (4 total) with light beam detectors spaced at intervals of 5, 30, and 50 mm away from the tip of the probe’s camera parallel to the optical imaging axis. These working distances were selected to demonstrate the range of possible magnifications achievable with the Pocket Colposcope. The light intensity patterns on these detectors provide an illustration of the cross-sectional beam shape as a function of working distance. The beam shapes were plotted with the x- and y-axis as the dimensions of the detector, and the z-axis was plotted as a heat map color-coded for optical power. Horizontal line scans through the center of beam shapes were used to characterize the homogeneity of the beam pattern at the various working distances. The reflector angle that provided the highest total optical power and beam pattern homogeneity at the 3 working distances was rapidly prototyped using a medical grade Acrylonitrile butadiene styrene plastic extrusion printer (Dimension 1200es Stratasys Inc., Eden Prairie MN). The inner reflector was polished with sandpaper, and silver metallic paint was applied to the smooth inner surface.

### Image quality and illumination characterization of the Generation 3 and 4 Pocket Colposcopes

Image quality was characterized using methods previously described and shown in Table 1 [11]. At least 5 sets of images for each imaging target listed in Table 1 were captured and analyzed per system. The USAF1951 resolution target images and the depth of field target images were analyzed with the open-source Fiji [19] software package. The SFRplus distortion target and Rezchecker color accuracy target were processed with the Imatest (Imatest, Boulder CO) software package [20]. The simulated cervix mannequin images were analyzed with Matlab (Mathworks, Natick MA) software packages.

**Table 1:** Summary quantitative image quality and illumination metrics and test targets.

Equation(#)	Metric	Formula	Test Target
**1**	Minimal Resolvable Feature Size (micron)	$\frac{1000}{2^{\left(Group\#+1+\frac{Element\#-1}{6}\right)}}$, Each group of Ronchi rulings consists of six elements, numbered from 1 to 6 (large to small physical size). The group and element of the smallest discernable set will provide resolvable feature size.	USAF 1951 (#R3L3S1P;Thorlabs Inc., Newton NJ)
**2**	Diagonal Field of View (mm)	$\sqrt{\begin{array}{l} {\left[\left(\frac{\text{Total}\;\#\;\text{of}\;\text{Horizontal}\;\text{Detector}}{\text{Horizontal}\;\text{Line}\;\text{Length}})*(\frac{2.5\text{mm}}{2^{\left(Group\#+\frac{Element\#-1}{6}\right)}}\right)\right]}^{2}\\ +{\left[\left(\frac{\text{Total}\;\#\;\text{of}\;\text{Vertical}\;\text{Detector}}{\text{Vertical}\;\text{Line}\;\text{Length}})*(\frac{2.5\text{mm}}{2^{\left(Group\#+\frac{Element\#-1}{6}\right)}}\right)\right]}^{2} \end{array}}$, The Total # of Pixels in the Horizontal Detector axis = 2592, the Total # of Pixels in the Vertical Detector axis = 1944, the Horizontal Line Length = width in pixels of the selected vertical Ronchi ruling, the Vertical Line Length = width in pixels of the selected horizontal Ronchi ruling. The Group and Element correspond to the selected Ronchi ruling.	
**3**	Depth of Focus (mm)	A 45° angled face resolution target with horizontal and vertical lines at a frequency of 5 line pairs per mm, corresponding to a feature size of 31.25 μm.	5-15DOF (#54–440, Edmund Optics, Barrington NJ)
**4**	Standard Mobile Imaging Architecture (SMIA) Television Distortion (%)	$\frac{\left(A-B\right)}{B}*100\%$ and $A=\frac{A_{1}-A_{2}}{2}$, where ***A***_***1***_ and ***A***_***2***_ are the outer side lengths of a square and ***B*** is the distance between the midpoints of the sides of the square ***A***_***1***_ and ***A***_***2***_. “+” = pincushion distortion and “-” = barrel distortion	SFRplus #CoG (imatest LLC., Boulder CO)
**5a**	Color Reproduction Error with Luminance Difference	**$\Delta E_{ab}^{*}=\sqrt{\left({\left(\Delta L^{*}\right)}^{2}+{\left(\Delta a^{*}\right)}^{2}+{\left(\Delta b^{*}\right)}^{2}\right)}$, L*** for lightness (or luminosity in the z axis), and x- and y-axis are cube root transforms of color data in a* and b*, color-opponent dimensions	X-Rite Rez Checker (#87–422; Edmund Optics, Barrington NJ)
**5b**	Color Reproduction Error	$\Delta C_{ab}^{*}=\sqrt{\left({\left(\Delta a^{*}\right)}^{2}+{\left(\Delta b^{*}\right)}^{2}\right)}$, x- and y-axis are cube root transforms of color data in a* and b*, color-opponent dimensions	
**6**	Percentage Specular Reflection	$\frac{\#\;\text{of}\;\text{saturated}\;\text{pixels}\;}{\text{total}\;\#\;\text{of}\;\text{pixels}}*100\%$, where a saturated pixel defined as pixel intensity ≥ 250.	#s504 Zoe (Gaumard Scientific, Miami FL)
**7**	Weber’s Contrast Ratio	$C_{W}=\frac{I_{B}-I_{F}}{I_{B}}$, *I*_*B*_ = Background Pixel Intensity *I*_*F*_ = Foreground Pixel Intensity	
**8**	Thermal Safety	IEC 60601–1 temperature limit of <48°C after 1 hour continuous operation	N/A

Minimal threshold used for our study was *C*_*W*_ ≥ 0.5.

An integrating sphere coated with 99% diffuse reflective Spectralon (Labsphere Inc., North Sutton NH) was used to assess the illumination source optical power and spectra as previously described [11]. In addition, the device was tested using an infrared non-contact thermometer (#62 Max+; Fluke Inc., Everett WA) and a short-wave infrared thermal imager (#Compact; SEEK Inc., Santa Barbara CA) to ensure that the device temperature did not exceed the International Electrotechnical Commission’s (IEC) 60601–1 limits for medical equipment in direct skin contact for less than 10 minutes of duration (48°C).

### Design implementations to ensure waterproof and anti-fog capabilities of the Generation 3 and 4 Pocket Colposcopes

We used a protective, antireflection coated, hydrophobic window (#88–356; Edmund Optics, Barrington NJ) for the Generation 3 and 4 Pocket Colposcopes. The hydrophobic window was placed into a custom computer numerical control (CNC) machine milled polycarbonate (1/16” thick) LED diffuser window, which has a lip machined out to hold the window in place and channels for each LED in the Generation 4 Pocket Colposcope. The hydrophobic window was bonded to the three dimensional printed plastic clamshell handles, sealed with medical grade epoxy, and surrounded with heat shrink tubing to aid in waterproofing the device. A similar construction process was used for the Generation 3 Pocket Colposcope, with the addition of crossed linear polarizers into the optical imaging pathway as previously described[11]. This waterproof optical imaging barrier eliminated the need to use anti-fog wipes prior to each procedure [21].

The imaging performance of the hydrophobic optical window was evaluated in a simulated moisture rich environment by comparing it to a bare uncoated protective glass protective window (#83–359; Edmund Optics, Barrington NJ) and a protective glass window pre-treated with commercial anti-fog wipe (Bausch & Lomb Fogshield XP). Three working prototypes were constructed with the different window configurations and misted with a dark green food coloring dye (FD&C Green No. 3). They were used to image a cervix phantom and cleaned with 70% isopropyl solution and lens wipe, and this was repeated 3 times. The commercial anti-fog wipe was reapplied after each cleaning as required by the manufacturer. These images were randomized and qualitatively scored by a blinded highly trained colposcopist from a scale of 0 to 10, where best image quality = “10” and the poorest image quality = “0”. A control set of images (no misting) was also included. The mean ± standard deviation of the image quality score for each system was calculated. Statistical differences between groups were assessed using one-way analysis of variance (ANOVA).

### Clinical evaluation

A clinical study in human subjects (n = 56) was conducted to compare the redesigned Pocket Colposcopes to a standard-of-care digital colposcope (Leisegang Optik 2) under Duke University Medical Center’s institutional review board (IRB) approved protocol, consent process, and data storage system (Pro00008173). The clinical study protocol was followed in the exact same fashion when evaluating the Generation 3 and Generation 4 Pocket Colposcopes. Since each generation of the Pocket Colposcope was being compared to the performance of the standard-of-care colposcope, the Generation 3 and Generation 4 Pocket Colposcope clinical studies were not performed on the same patients to limit the number of colposcopy devices used per patient to two. Adult subjects (18–65 years of age) undergoing routine colposcopy and/or Loop Electrosurgical Excision Procedure (LEEP) treatment provided written informed consent for sequential imaging with the Pocket Colposcope and standard-of-care colposcope. The consent process, eligibility criteria check list, and original consent form were securely stored within a locked office that is accessible only to protocol approved personnel. All data was de-identified, and a new study randomized study identification number was generated before being stored on an encrypted and password protected server database, Research Electronic Data Capture Platform (REDCap), maintained by the Duke Translational Medicine Institute [22].

As part of the standard-of-care examination procedure, a speculum was placed and any blood or mucous was removed from the cervix using a large tipped cotton or rayon swab. A 5% acetic acid solution was then applied to the cervix, followed by digital image capture with the order of systems randomized between the standard-of-care colposcope (3.75X Magnification) and the Pocket Colposcope (6X magnification). Acetic acid was always reapplied to the cervix, prior to image capture with each colposcope. All clinical decisions were completed using the standard-of-care colposcope, including direct biopsy or LEEP. Cervix specimens were processed and read by institutional pathologists as the gold-standard diagnostic reference [23].

A highly trained colposcopist reviewed randomized image panels for the Pocket Colposcope and standard-of-care colposcope and provided a diagnosis for each image. The images were classified as normal, grade 1, and grade 2, or cancer based on visual inspection of features. Panels were randomized such that concordant images were not in the same set; the clinician was blinded to the patient’s history and pathological results. The REDCap database enabled secure storage of clinician’s interpretations of the randomized image panels, through a web-based portal. A statistical software package (Stata 13.1 MP; STATA Corp, College Station TX) was used to assess primary outcome measures, which include the level of agreement (%) and ***κ*** (*kappa)* statistic between systems. The secondary outcome measures, include the level of agreement (%) and ***κ*** (*kappa)* statistic between each system and pathology. When comparing each system to pathology, cervical intraepithelial neoplasia (CIN1) or a higher histopathology diagnosis was used as our binary cutoff.

## Results

### Crossed-Polarization design for Generation 3, reflector design and optimization for Generation 4

**Fig 3:**
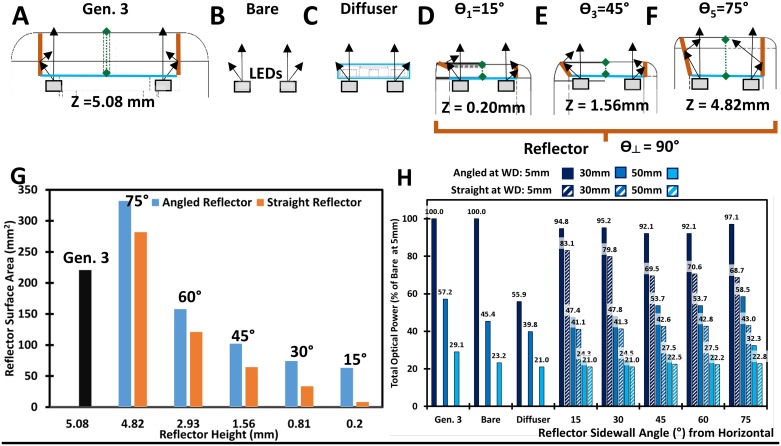
Representative graphical simulations of differences in light ray path, beam shape, and beam intensity between reflector designs with varying sidewall angles using the integrated white LEDs. Zemax Ray Tracing and Solidworks digital drafting programs were used to simulate the effect if any of different integrated reflector cone designs with the Generation 4 Pocket Colposcope. Simplified diagrams of simulation set up are shown for (A) Generation 3, (B) Bare LEDs, (C) Diffuser Window, (D) Reflector at 15° from the indicated axis (perpendicular to the imaging axis) (E) Reflector at 45°from indicated axis, and (F) Reflector at 75° from indicated axis. The surface area of the reflectors is shown as a function of height and angle and the total optical power for a working distance of 5, 30 and 50 mm (G). Angling the reflector (light blue) netted a gain of 20–30% surface area when compared to the matched height straight reflector (orange). Similarly, (H) we noted that the 75° angled reflector performed the best out of all angled (solid blues) or straight (hatched blues) reflectors, at all working distances. For both Pocket Colposcopes the expected field of view is roughly equivalent to the respective working distance.

Simulations were carried out for six different tip designs (Fig 3A–3F) (one design for the Generation 3 device and five different designs for the Generation 4 device). The Generation 4 Pocket Colposcope with reflector surface at a 75° angle and reflector height of 4.82 mm with respect to the vertical axis (which is perpendicular to the optical imaging axis, in Fig 3F) provided the greatest recovery of optical power and the most homogenous beam pattern across our target working distances of 5, 30, and 50 mm. Increasing the angle with respect to the imaging axis, generally improved beam uniformity and total optical power across all working distances (Fig 3H). Note the gradual improvement in “tightness” of the beam cutoff at the 5 mm working distance across the different designs (S1 Fig). We also observed that the angled reflector when compared to the matched height straight reflector netted a 20–25% increase in surface area (Fig 3G). We also noted that the straight height reflector tended to introduce distinct illumination hot spots when compared to the matched height angled reflector (see S1 Fig).

### Image quality and illumination characterization of the Generation 3 and 4 Pocket Colposcopes

The Pocket Colposcope system’s quantitative image quality performance did not significantly change between Generation 3 and Generation 4 designs (see Table 2 and S2 Fig). The Pocket Colposcope system has the capability to resolve features as small as 9 μm when compared to the 21 μm resolution of the standard-of-care colposcope at its highest respective magnification setting. The diagonal field of view (FOV) is smaller for the Pocket Colposcopes when compared to the standard-of-care across all magnifications, but still provides adequate field of view for 4 quadrant surveillance of the cervix at the low magnifications. The Pocket Colposcopes could not match the depth of focus of the standard-of-care system at the lowest magnification, but performed more comparably at the intermediate magnification, which is most commonly used by providers. The level of distortion is minor <1% for the standard-of-care at all magnifications and <2% for the Pocket Colposcopes. The mean color accuracy error ΔC* was ~4.2 and ΔE* was ~8.4 for the Pocket Colposcope systems, while the standard-of-care’s color accuracy error was slightly lower at ΔC* = 2.9 and ΔE* = 5.24. The removal of the cross-polarizers and reflector design in the Generation 4 system further lowered our electrical power requirements by a factor of 6 when compared to the Generation 3 system (Table 2), but can still provide adequate illumination of the cervix by redirecting light normally lost at the outer edges of the beam pattern. The temperature of the Pocket Colposcopes were monitored over the course of an hour. The Generation 4 Pocket Colposcope was on average 12°C cooler than the Generation 3 system after 1 hour of continuous operation (42° vs. 29°C), S3 Fig.

**Table 2:** Summary of illumination and imaging characteristics demonstrating comparable performance between systems across a range of working distances or magnification settings.

Working Distance (mm)	7			30			50			SMIA Distortion	Mean Color Accuracy		Mean Probe Temperature after 1hr continous operation	Electrical Power	Optical Power	Beam Diameter
Metrics	RFS	DFOV	DOF	RFS	DFOV	DOF	RFS	DFOV	DOF							
Units	μm	mm	mm	μm	mm	mm	μm	mm	mm	%	ΔC*_ab_	ΔE*_ab_	C	W	μW	mm
**Generation 3 Pocket Colposcope**	**7.8**	**7.5**	**1.1**	**22.1**	**35.7**	**9.5**	**50.6**	**51.7**	**10.3**	**-1.80**	**4.28**	**8.48**	**41°**	**1.24**	187.9±10.3*	40.2±1.9*
**Generation 4 Pocket Colposcope**	**8.8**	**8.4**	**1.2**	**24.8**	**30.7**	**8.5**	**49.5**	**51.3**	**10.9**	**-1.19**	**4.16**	**8.31**	**29°**	**0.5**	37.9±6.7*	33.8±2.3*
**Magnification at WD = 300 mm**	**15X**			**7.5X**			**3.75X**									
**Standard-Of-Care Colposcope**	**20.9**	**19.4**	**7.05**	**30.7**	**38.3**	**11.2**	**50.6**	**76.9**	**19.6**	**-0.60**	**2.9**	**5.24**	**N/A**	**18.0**	**1301.2±84.5**	**62.1±0.7**

***RFS*** is Resolvable Feature Size, ***DFOV*** is Diagonal Field of View, ***DOF*** is Depth of Focus, ***SMIA*** is Standard Mobile Imaging Architecture Distortion. ΔE*_ab_ color error acounting for any luminance difference and ΔC*_ab_ does not account for luminance. Values are presented as mean ± standard deviation of illumination electrical power (W), optical power (μW), and beam diameter (mm) from n = 5 measures using our previously described testing station.

“*” indicates 2 tailed paired t-test and one-way ANOVA p<0.01 when compared to standard-of-care colposcope.

### Design implementations to ensure waterproof and anti-fog capabilities of the Generation 3 and 4 Pocket Colposcopes

**Fig 4:**
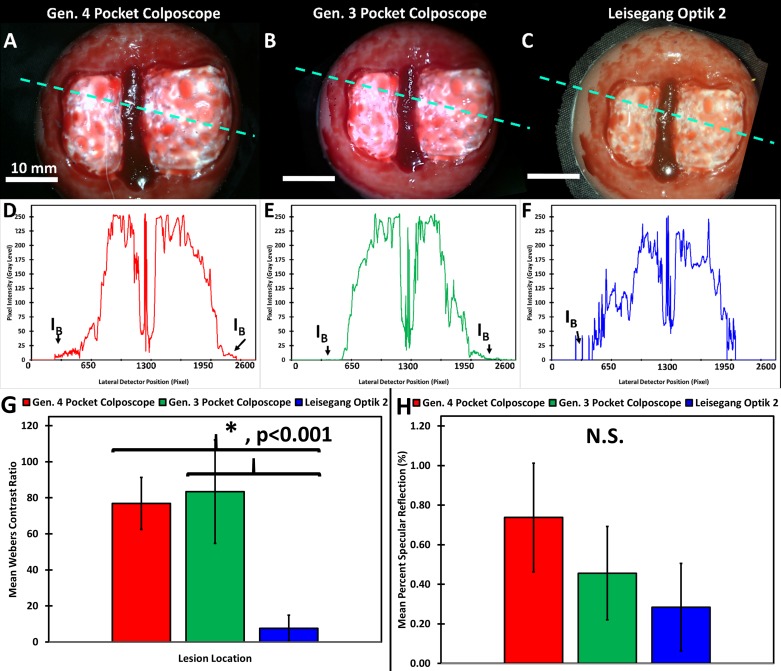
Improved contrast is observed with the Generation 4 Pocket Colposcope and Generation 3 Pocket Colposcope when compared to standard-of-care colposcope in a high-grade cervical mannequin lesion. Representative images taken of a high-grade mannequin cervical lesion in a custom dark chamber with Generation 4 Pocket Colposcope (A), Generation 3 Pocket Colposcope (B), and standard-of-care colposcope(C). The scale bars are 10 mm (ABC). A representative horizontal line scan is shown to assess the level of contrast of the simulated lesions across systems (DEF). These horizontal scans were repeated 5 times on 5 repeated image captures to calculate the mean Weber’s contrast ratio and standard deviation, which are shown in (G). The mean Weber’s Contrasts were significantly higher than the standard-of-care colposcope for both generations of the Pocket Colposcope with p<0.0001, by 2-sample t-test and one-way ANOVA. However, there was not a significant difference between the generations of the Pocket Colposcope. The level of specular reflection was not significantly different between the systems with one-way ANOVA p-value >0.1 and 2-sample t-test p-values > 0.06. The percent specular reflection was calculated from 5 repeated image captures and the mean and standard deviation are shown in (H) with no significant differences from the one-way ANOVA or between group t-tests.

Representative images of a simulated high grade lesion were captured using each system (Fig 4). The mean Weber’s contrast was comparable (Fig 4D and 4E) between the Generation 4 Pocket Colposcope and Generation 3 Pocket Colposcopes. Both generations of the Pocket Colposcope had significantly higher Weber’s Contrast when compared to the standard-of-care colposcope, p<0.001 for one-way ANOVA and 2-sample t-test (Fig 4G). The level of specular reflection was not significantly different between the systems with one-way ANOVA p-value >0.1 and 2-sample t-test p-values > 0.06 (Fig 4H).

**Fig 5:**
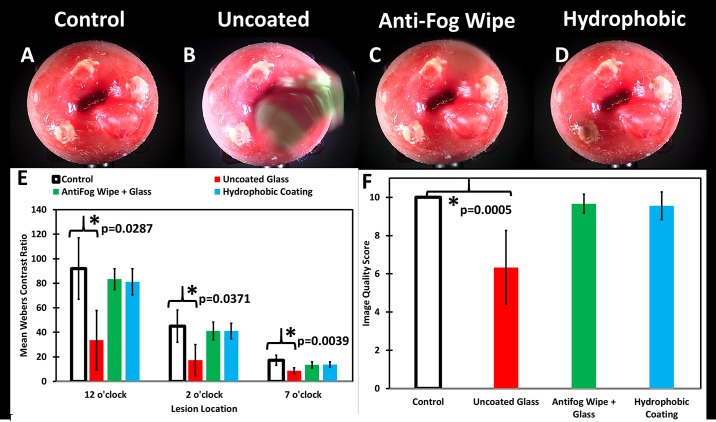
Representative cervical mannequin images misted with green food dye with varying treatments. with qualitative image quality assessed by 3 blinded clinicians, and quantitative image quality by computing Weber’s contrast. The cervix mannequin has acetowhitened lesions at 12, 3, and 7 o’clock positions and a cyst at the 4 o’clock position. Control untreated glass optical window (A), dye misted untreated glass optical window (B), dye misted anti-fog wipe treated glass window (C), and dye misted hydrophobic window (D). (E) Weber’s contrast values were calculated for each lesion (n = 3) and the mean and standard deviation were determined from 3 repeated images of the same cervix. The uncoated glass (red) performed significantly worse than the control (white) at all 3 lesion positions (p<0.02 for all using 2-sample t-test and one-way ANOVA). The anti-fog wipe (green) and hydrophobic window (light blue) were not significantly different from the control group (p>0.1). (F) Qualitative assessment by 3 blinded clinicians of each treatment and control group noted a significant degradation in image quality for the uncoated glass that was misted with dye when compared to control (p<0.005).

Next, the ability of a hydrophobic window and single use anti-fog wipes to prevent potential condensation or liquid film formation was assessed. Both approaches were compared to uncoated glass and a non-misted bare imager, which served as a control. The mean Weber’s contrast for all 3 lesions in the uncoated glass group was significantly depressed when compared to that of the control group (Fig 5). The Weber’s contrast values for the anti-fog wipe treated window and hydrophobic window were not significantly lower than that of the control group (Fig 5E). There was a significant difference in the perceived image quality score between the misted uncoated glass and control groups (Fig 5F).

### Clinical evaluation

After demonstrating comparable image quality and safety using standardized benchmarks, the Generation 3 and 4 Pocket Colposcopes were compared to each other and to a standard-of-care colposcope, in a pilot clinical study. Due to the small number of patients in the pilot clinical study, several of the baseline demographics of the patient population enrolled in our clinical study differed between our device groups. These variables including ethnicity, high-risk HPV positive status, prior Pap smear classification, procedure type, and pathology outcome (Table 3). Ethnicity was different between device groups, with 8.3% self-identifying as Hispanic in the Generation 3 device group and only 3.1% self-identifying as Hispanic in the Generation 4 group, with a p = 0.0009 from a Fisher’s Exact test. High-risk HPV positive statuses were higher in the Generation 4 group at 16.1% versus 4.2% in the Generation 3 group, with p = 0.002, Table 3. Pap smear classification was significantly different between system generations, with 4.2% of Generation 3 subjects having ASCUS versus 36.7% in Generation 4 (p = 0.0006, Table 3). The procedure type was also significantly different with the majority (66.7%) of Generation 3 subjects undergoing LEEP versus only 21.9% of Generation 4 subjects, (p = 0.001). This referral bias lead to a significantly different pathology distribution between generations with 54.2% with CIN2+ in Generation 3 and only 31.9% with CIN2+ in Generation 4 (p = 0.048, Table 3).

**Table 3:** Demographic characteristics of study population across devices investigated.

		Generation 3 (n = 24)		Generation 4 (n = 32)		Total (n = 56)		
		Mean±SD		Mean±SD		Mean±SD		p-value
**Age (years)**		32.0±8.68		36.1±10.2		34.4±9.70		0.125^+^
		**%**	**#**	**%**	**#**	**%**	**#**	
**Ethnicity**	Hispanic	8.3	2	3.1	1	5.4	3	0.0009*
	Not Hispanic	0	0	59.4	19	33.9	19	
	Unknown	91.7	22	37.5	12	60.7	34	
**Race**	White	29.2	7	37.5	12	33.9	19	0.863*
	Black or African American	58.3	14	50.0	16	53.6	30	
	Unknown	12.5	3	12.5	4	12.5	7	
**HIV + Status**	Positive	0	0	0	0	0	0	0.063*
	Negative	4.2	1	25.0	8	16.1	9	
	Unknown	95.8	23	75.0	24	83.9	47	
**High Risk HPV + Status**	Positive	4.2	1	16.1	5	10.7	6	0.002*
	Negative	12.5	3	48.4	15	32.1	18	
	Unknown	83.3	20	35.5	12	57.1	32	
**Pap Smear Classification**	Normal	0	0	3.3	1	1.8	1	0.006*
	ASCUS	4.2	1	34.3	11	21.4	12	
	ASC-H	12.5	3	12.5	4	12.5	7	
	LSIL	20.8	5	28.1	9	25.0	14	
	HSIL	41.7	10	12.5	4	25.0	14	
	AGC	4.2	1	0.0	0	1.8	1	
	Unknown	16.7	4	9.4	3	12.5	7	
**Procedure Type**	Colposcopy	33.3	8	78.1	25	58.9	33	**0.001**^**x**^
	LEEP	66.7	16	21.9	7	41.1	23	
**Pathology**	Normal	16.7	4	56.3	18	39.3	22	**0.048**^**x**^
	CIN1	25.0	6	12.5	4	17.9	10	
	CIN2+	58.3	14	31.3	10	42.9	24	

The “+” symbol indicates that a two independent sample t-test was performed to assess the interval dependent variable as stratified by device generations. The “*” symbol indicates that a Fisher’s Exact Tests was performed to assess the categorical variables with elements having frequency of 5 or less, as stratified by device generation. The “^x^” symbol indicates Chi Square Tests were performed to assess the categorical variables with elements having frequency greater than 5, as stratified by device generation. The Human Papilloma Virus (HPV) screening test used at our institution detects the presence any of the following high risk genotypes: 16, 18, 31, 33, 35, 39, 45, 51, 54, 56, 58, 59, 66, 68; but does not provide specific typing. The abnormal Papanicolaou results are stratified by the following classifications: Atypical Squamous Cells of Undetermined Significance (ASCUS), Atypical Squamous Cells, cannot exclude HSIL (ASC-H), Low-grade Squamous Intraepithelial Lesion (LSIL), High-grade Squamous Intraepithelial Lesion (HSIL), Atypical Glandular Cells (AGC). The abnormal results from colposcopy guided biopsy and LEEP specimens are stratified by the following classifications: Cervical Intraepithelial Lesion (CIN) and numeric level. For our analysis we combined CIN2 and CIN3 into combined category of CIN2+.

**Fig 6:**
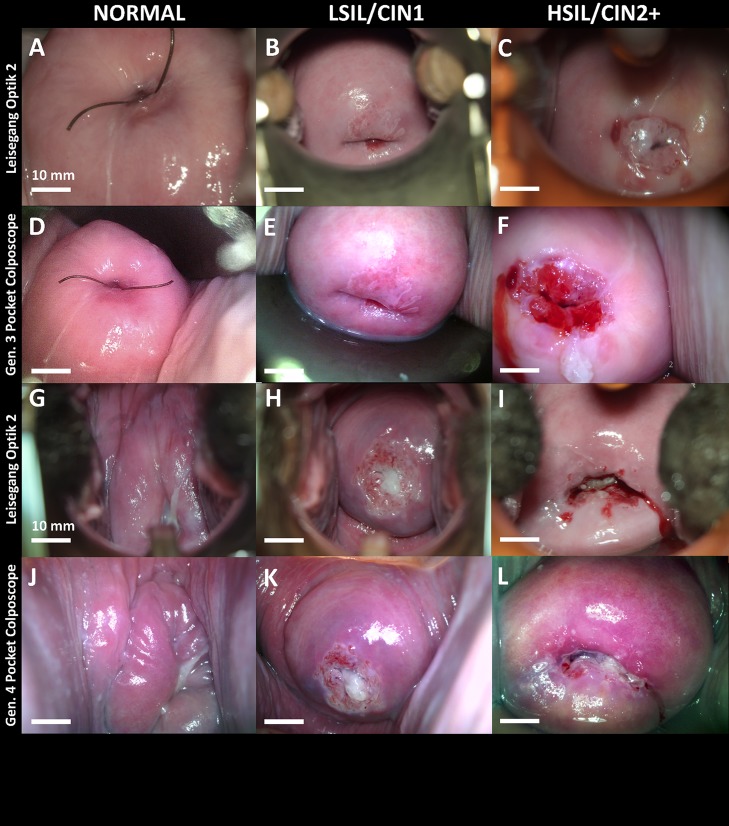
Representative images stratified by biopsy confirmed histopathology captured by a standard-of-care colposcope, and the Generation 3 and Generation 4 Pocket Colposcopes. Reference images were captured with a standard-of-care colposcope at 3.75X magnification at a working distance of 300 mm (ABC, GHI); Generation 4 Pocket Colposcope (DEF) at 30 mm working distance; and Generation 3 Pocket Colposcope (JKL). The scale bars are 10 mm. Pathology confirmed diagnosis were Normal (A, D, G, J), CIN1 (B, E, H, K), and CIN2+ (C, F, I, L).

Representative cervical images captured with the Generation 3 (Fig 6D, 6E and 6F) and the Generation 4 (Fig 6J, 6K and 6L). The Pocket Colposcopes demonstrate a comparable field of view and quality, with respect to the standard-of-care captured images (Fig 6A, 6B, 6C, 6G, 6H and 6I). The images are stratified in columns by histopathology confirmed classification, with the first column consisting of Normal (Fig 6A, 6D, 6G and 6J), CIN1 (Fig 6B, 6E, 6H and 6K), and CIN2+ (Fig 6C, 6F, 6I and 6L).

The overall level of agreement between our Generation 4 Pocket colposcope with the reflector and the standard-of-care colposcope was 75.0% with *κ* value of 0.4941 and p = 0.0024, when using a Grade 1 or higher diagnostic cutoff (Grade 1+ interpretations were binned together as positive). This level of agreement was comparable to that of our Generation 3 level of agreement with the standard-of-care colposcope, at 75.0% with *κ* value of 0.4000 and p = 0.028, when using a Grade 1+ diagnostic cutoff. The true positive and true negative level of agreement and kappa statistic as stratified between Generation 3 and Generation 4 Pocket Colposcope in Table 4.

**Table 4:** Summary of level of agreement and kappa statistic between Pocket Colposcope and standard-of-care colposcope (clinical image interpretations) stratified by true positive (Grade 1+) and true negative (Grade 1-).

		**Standard-Of-Care** **Colposcope**	**kappa** **(p-value)**
	**Interpretation**	**Grade** **1+**	
**Generation 3 Pocket** **Colposcope**	**Grade** **1+**	87.5 (14)	**0.4000** **(0.0228)**
**Generation 4 Pocket** **Colposcope**	**Grade** **1+**	70.0 (14)	**0.4375** **(0.0059)**
	**Interpretation**	**Grade** **1-**	
**Generation 3 Pocket** **Colposcope**	**Grade** **1-**	50.0 (4)	-
**Generation 4 Pocket** **Colposcope**	**Grade** **1-**	83.3 (10)	-

The overall level of agreement between our Generation 4 Pocket Colposcope and pathology was 62.5% with *κ* value of 0.2500 and p = 0.077, when using a VIA+ diagnostic cut-off (Grade 1+ interpretations were binned together as positive). The matched level of agreement between standard-of-care colposcope and pathology was 59.4% and was not significantly different when compared to matched Pocket Colposcope’s performance (Pearson chi-square p = 0.18). The overall level of agreement between our Generation 3 Pocket Colposcope and pathology was 66.6% with *κ* value of 0.1420 and p = 0.2193, when using a VIA+ diagnostic cut-off. The matched level of agreement between standard-of-care colposcope and pathology when using a VIA+ diagnostic cut-off was 75.0%, which was not significantly different to the matched Pocket Colposcope’s performance (Pearson chi-square p = 0.15).

## Discussion

The World Health Organization’s recommends a “see and treat” paradigm for cervical cancer prevention in low and middle-income countries using VIA (if HPV screening is not available) and cryotherapy. VIA has several limitations including poor specificity, high inter-observer variability, and lack of image capture [2]. Thus, there is an unmet need for a low-cost portable colposcope that could address several of these limitations and improve women’s access to cervical cancer screening. This study describes our strategy to develop a solution with comparable image quality to standard-of-care digital colposcopes and compatible with liquid chemical cleaning procedures used in the field. The primary goals of this study were to improve image contrast levels, increase the illumination beam pattern uniformity, reduce the required electrical power budget of our system, improve anti-fogging and incorporating water resistant features into our device, and enhance system portability.

Our strategy to increase illumination and collection efficiency of the Pocket Colposcope was to utilize a reshaped illumination beam with an integrated reflector. Our ability to minimize specular reflection without the use of cross-polarizers stems from a combination of reducing stray light and improved beam collimation and uniformity. The use of computer aided three-dimensional modeling and ray tracing simulations [24] allowed us to perform virtual design optimization of the reflector geometry by reducing the amount of time and resources required for iterative prototyping and experimental validation. Our simulations indicated that the tallest (4.82 mm) and steepest angled reflector (75°) yielded the highest beam uniformity and optical power at our target range of working distances. Specifically, the angled reflector provides the largest increase in surface area, while minimizing illumination hot spots when compared to the matched straight or vertical reflector tube of equal height. Our quantitative image quality comparisons for field of view, resolving power, and color accuracy did not result in any significant difference between the two generations of Pocket Colposcopes and the standard-of-care digital colposcope. The Generation 3 and 4 Pocket Colposcopes provided a 2 to 3-fold higher Weber’s contrast ratio (70–80) compared to the standard-of-care colposcope (30). The required electrical power budget was reduced to 1/7 that of the Standard-of-care for the Generation 3 Pocket Colposcope and 1/35 that of the standard-of-care colposcope; which are attributable to the removal of the crossed polarizers and reflector implementation found in the Generation 4 Pocket Colposcope. The elimination of the cross-polarizers and the integration of the reflector eliminate the need for an external control box used in our Generation 3 Pocket Colposcope [11], lowering the required electrical power budget. Further, the number of external optical elements has been simplified from 5 to 3 in the Generation 4 system when compared to the Generation 3 system, leading to further reduced material and assembly costs. Illumination improvements could be achieved by reshaping the beam pattern and enhancing beam uniformity with an integrated reflector. The removal of illumination hot spots should improve precancerous lesion contrast as demonstrated by our mannequin cervix imaging studies. However, this design iteration comes at the cost of eliminating the green LEDs in the Generation 4 Pocket Colposcope. The plan in future design iterations is to integrate back the green LEDs without the need for a voltage booster thereby maximizing the benefits of Generation 3 and Generation 4 Pocket Colposcopes into one system using the new insights we have gained from the addition of the reflective surface at the tip of the device. An added benefit of the improved lighting efficiency was a decreased thermal heating risk to patients, as both of our systems successfully met the IEC 60601–1 temperature limit of <48°C for a medical device in direct contact with human skin for <10 minutes and the more stringent ≤43°C limit direct skin contact ≥ 10 minutes thresholds [25]. The Generation 4 Pocket Colposcope was on average 6–14°C cooler over a period of 1-hour continuous operation than the Generation 3 Pocket Colposcope, reducing any potential patient discomfort due tissue heating by the device.

The hydrophobic window design allows us to develop a more robust probe that is compatible with chemical immersion sterilization most often used in resource limited settings [26]. The new design is also better suited for implementation in resource-limited settings due to the reduced consumable cost of anti-fog wipes when compared to the Generation 1–2 Pocket Colposcope. Furthermore, Generation 1–2 required sterilization with Ethylene Oxide gas not readily available in all Low and Middle Income Countries.

Our pilot clinical evaluation of our Generation 4 Pocket Colposcope revealed a comparable level of agreement of 75.0% with unweighted *κ* value of 0.4941, p = 0.0042 with the standard-of-care colposcope. This level of agreement matched the Generation 3 system’s agreement (also 75.0%) with the standard-of-care colposcope. Qualitatively, the most important predictor variable for misdiagnosis with the Pocket Colposcope compared to the standard-of-care colposcope was lack of acetowhitening. This misclassification error between the Pocket Colposcope and standard-of-care colposcope can be explained by the unequal time delay occurring between the application of acetic acid and the subsequent image capture. Due to the temporal nature of the acetowhitening effect, we plan to incorporate the use of a timer in future protocols to ensure that an equal time passes after each application of acetic acid. Furthermore, illumination matching and digital processing to maintain white balance are important considerations for the Pocket Colposcope moving forward.

The transvaginal approach provides for some unique benefits and challenges when compared to traditional colposcopy outside of optical parameters. A traditional colposcope would typically be wiped down with a germicidal sheet after each patient. The Pocket Colposcope is placed within a speculum and can potentially come in contact with blood and mucous and therefore needs to undergo high-level disinfection. The Pocket Colposcope is first wiped down with a germicidal wipe for the gross removal of excessive mucous and/or blood. The device is then reprocessed with high level disinfectant through complete immersion in one of the following chemical agents Bleach, Cidex OPA (0.55% Ortho-phthalaldehyde), Hydrogen Peroxide[27,28]. A significant benefit from the transvaginal approach is the enhanced portability when compared to a traditional colposcope due to the miniaturization of system components and reduction of system size to fit within the speculum. Our device can readily fit into the medical professional’s coat pocket in a similar fashion to an otoscope or ophthalmoscope. Furthermore, the improved illumination efficiencies of the Pocket Colposcope allows its operation off the USB port of a laptop, tablet, or smartphone and eliminates the need to be tethered to a walled electrical outlet. The Pocket Colposcope can be readily steadied by resting on the lower or upper speculum blades by the provider. Thereby, eliminating the need for an external stand used by traditional colposcopes. Anecdotally, early users of the Pocket Colposcope have provided feedback requesting a way to initiate image capture and to select the modes of illumination directly on the device, to allow for a completely single handed operational experience. Our future work will focus on incorporating key improvements to the Pocket Colposcope’s ergonomics that include: (1) on device handle image capture button, (2) on device handle illumination color and intensity selection button, (3) miniaturization of illumination circuitry into the probe handle with both white and green illumination capability, (4) a simplified tactile sliding zoom control, (5) a more ergonomic angled handle to allow for less user strain when operating the device, and (6) an improved waterproofing technique involving plastic welding the probe’s clamshells together in lieu of medical-grade epoxy and heat shrink tubing currently employed in the Generation 3 and Generation 4 systems.

We have established that our Generation 4 Pocket Colposcope performs nearly identically to the prior Generation 3 Pocket Colposcope and standard-of-care colposcope, while eliminating the external control box and with improved hardiness required for use in resource-limited settings. The Generation 3 Pocket Colposcope’s had the benefits of longer operational time between charges (12 vs. 8 hours) and the retention of the green LEDs enabling improved diagnostic ability through enhanced detection of vascular changes associated with precancerous lesions. The more compact and portable Generation 4 Pocket Colposcope would be better suited for the primary care setting as an initial screening tool while the Generation 3 Pocket Colposcope, which has all the attributes of a traditional clinical colposcope will be well-suited for use in a traditional colposcopy setting. Both generations of the Pocket Colposcope could capture Lugol’s Iodine images as part of VILI (visual inspection with Lugol’s Iodine).

## Supporting information

S1 Fig Comparison of predicted beam patterns from Generation 4 angled and straight reflector designs at the 5 mm working distance. The simulations for the expected beam pattern for our Generation 4 revealed a cross shaped pattern for the bare LEDs (A), which improved to a circular projection with the diffuser in place (B). The edge of the circular patterns improved greatly with increasing reflector angle (CDE, 15 to 75°). Interestingly, the straight height reflectors introduced some distinct undesirable hot spots (red speckling) in the beam pattern at the taller heights (GH, 1.56 to 4.82 mm).
(TIF)

S2 Fig Comparable levels of image quality between generations of the Pocket Colposcope and standard-of-care digital colposcope. Representative target images captured by Generation 4 Pocket Colposcope (A, D, G, J), Generation 3 Pocket Colposcope (B, E, H, K) all taken at the 35 mm working distance, and Standard-of-care colposcope (C, F, I, L) set at captured at the 300 mm working distance with a magnification setting of 7.5X. The minimal resolvable feature of 22.1 microns was comparable between our systems (DE) and better than the high-end reference system (F) set at 31.6 microns. The depth of field was also comparable between our systems at ~5 mm (GH) for the 5 line pairs per mm horizontal depth target, but not as good as high-end system at 11 mm (I). The color reproduction error was measured using a NIST calibrated color target the Pocket Colposcope system (JK) which was slightly higher than the reference system (L).
(TIF)

S3 Fig Representative thermal imaging of the Gen 4. Pocket Colposcope reveals a net reduction in operating temperature of the reflector based system when compared to the prior Generation 3 Pocket Colposcope. Representative thermal images (long-wave infrared) of the Generation 3 Pocket Colposcope (A) and Generation 4 Pocket Colposcope (B), note the 6 to 14°C reduction in operating temperature with the more efficient reflector based system.
(TIF)

S1 Table A selected summary of key characteristics from a range of commercial colposcopes and our systems. (DOCX)
