# Supplementary figures and images for: An integrated strategy for improving contrast, durability, and portability of a Pocket Colposcope for cervical cancer screening and diagnosis
Source: PLoS One. 2018 Feb 9;13(2):e0192530. doi: 10.1371/journal.pone.0192530 (PMC5806883; doi:10.1371/journal.pone.0192530)

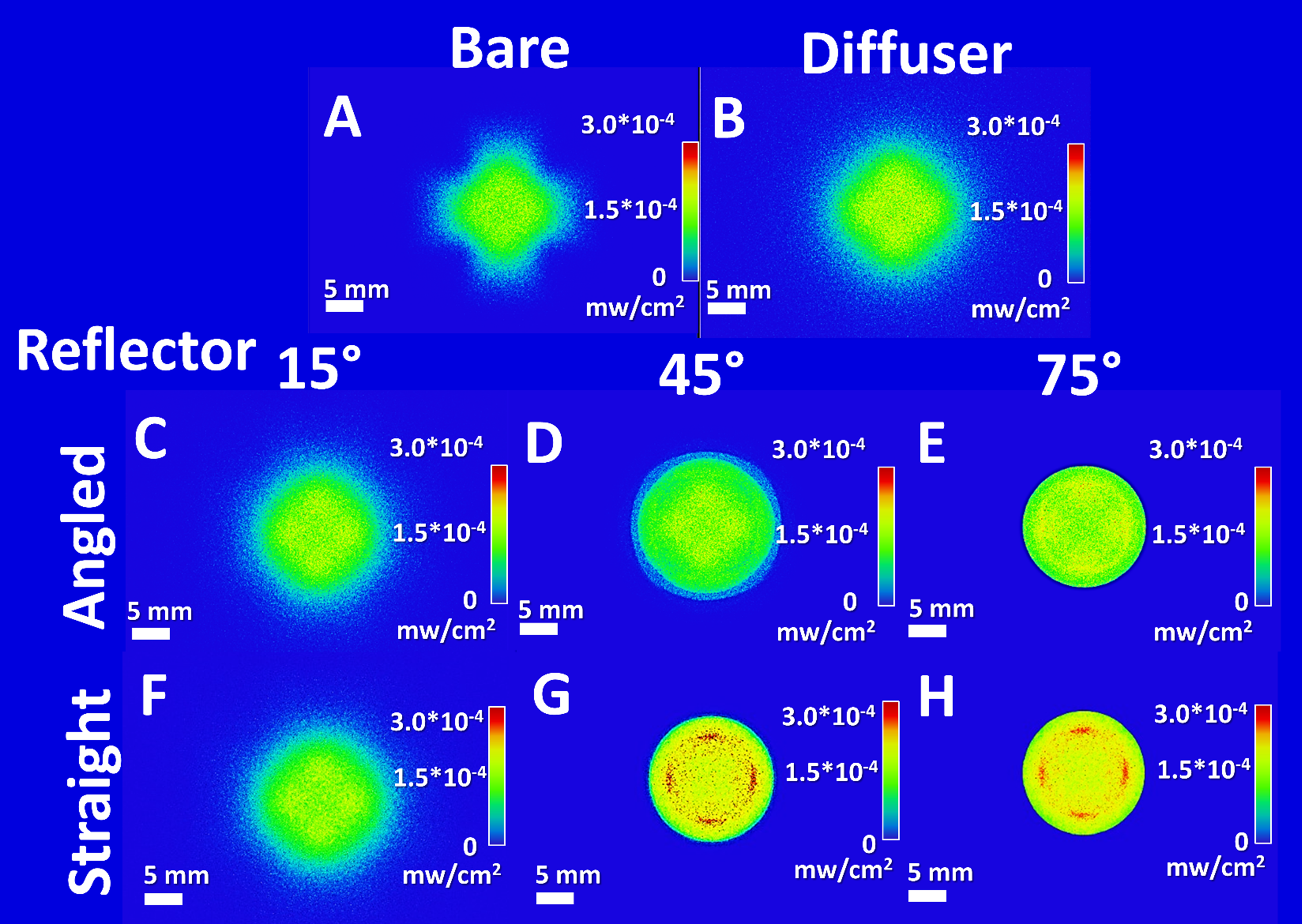

Supplement: S1 Fig — The simulations for the expected beam pattern for our Generation 4 revealed a cross shaped pattern for the bare LEDs (A), which improved to a circular projection with the diffuser in place (B). The edge of the circular patterns improved greatly with increasing reflector angle (CDE, 15 to 75°). Interestingly, the straight height reflectors introduced some distinct undesirable hot spots (red speckling) in the beam pattern at the taller heights (GH, 1.56 to 4.82 mm). (TIF) [file pone.0192530.s001.tif]

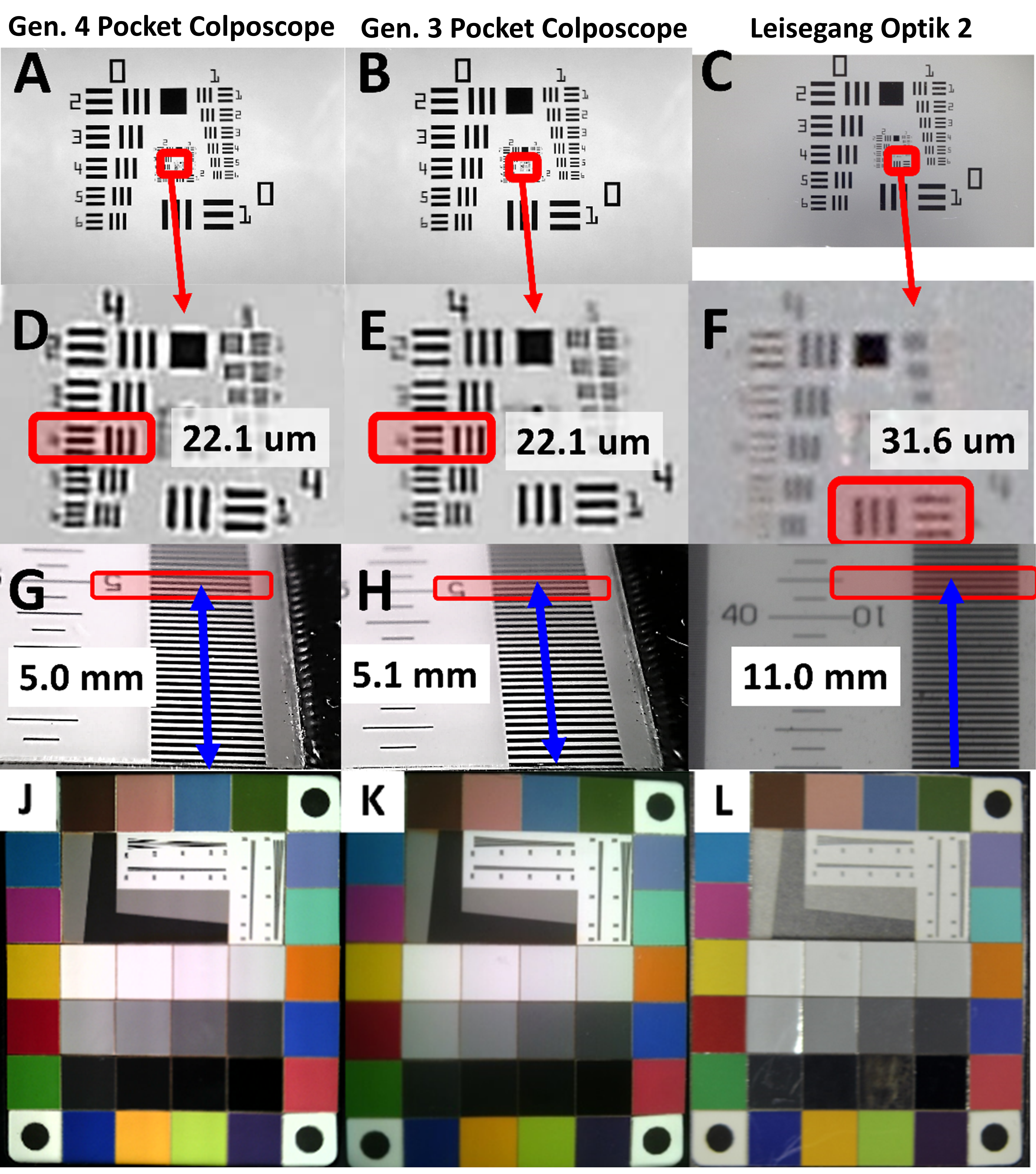

Supplement: S2 Fig — Representative target images captured by Generation 4 Pocket Colposcope (A, D, G, J), Generation 3 Pocket Colposcope (B, E, H, K) all taken at the 35 mm working distance, and Standard-of-care colposcope (C, F, I, L) set at captured at the 300 mm working distance with a magnification setting of 7.5X. The minimal resolvable feature of 22.1 microns was comparable between our systems (DE) and better than the high-end reference system (F) set at 31.6 microns. The depth of field was also comparable between our systems at ~5 mm (GH) for the 5 line pairs per mm horizontal depth target, but not as good as high-end system at 11 mm (I). The color reproduction error was measured using a NIST calibrated color target the Pocket Colposcope system (JK) which was slightly higher than the reference system (L). (TIF) [file pone.0192530.s002.tif]

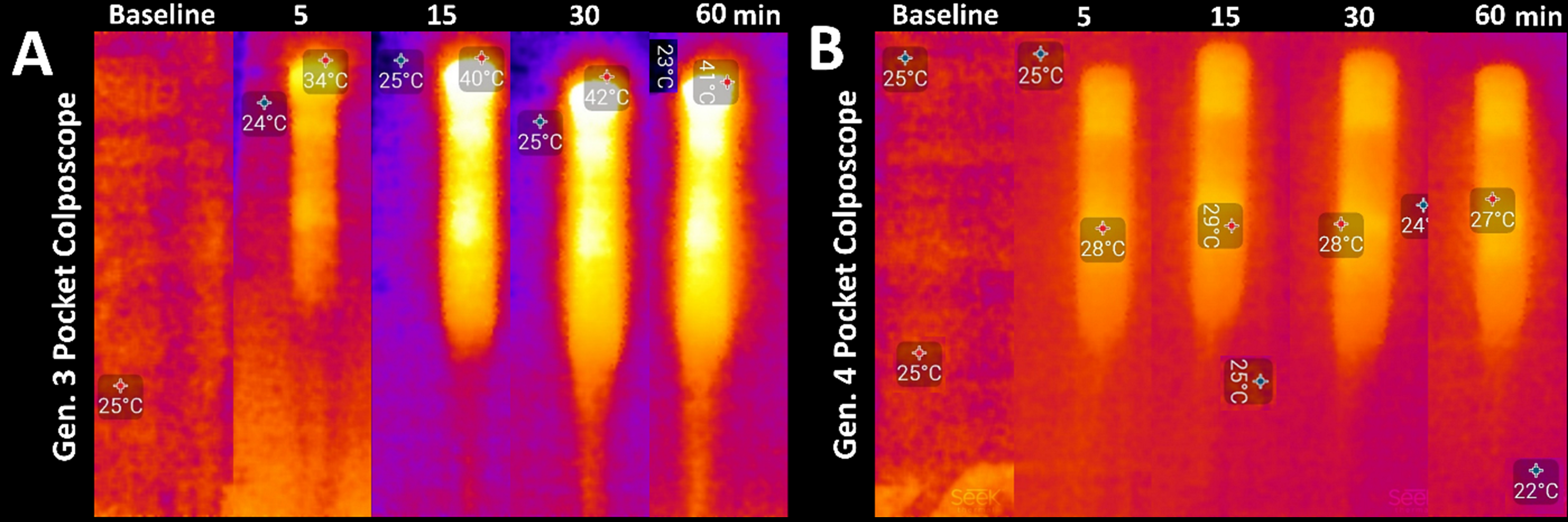

Supplement: S3 Fig — Representative thermal images (long-wave infrared) of the Generation 3 Pocket Colposcope (A) and Generation 4 Pocket Colposcope (B), note the 6 to 14°C reduction in operating temperature with the more efficient reflector based system. (TIF) [file pone.0192530.s003.tif]
