# Supplementary material for: An integrated strategy for improving contrast, durability, and portability of a Pocket Colposcope for cervical cancer screening and diagnosis
Source: PLoS One. 2018 Feb 9;13(2):e0192530. doi: 10.1371/journal.pone.0192530 (PMC5806883; doi:10.1371/journal.pone.0192530)
Supplement: S1 Table — (DOCX) [file pone.0192530.s004.docx]

**S1 Table: A selected summary of key characteristics from a range of commercial colposcopes and our systems**

| **Specifications** | | **Weight** | **Optical Magnification** | **Resolution** | **Field of View** | **Depth of Focus** | **Working Distance** | **Focus** | **Stability** | **Duration of Battery Operation** | **LED Illumination Characteristics** | | |
| --- | --- | --- | --- | --- | --- | --- | --- | --- | --- | --- | --- | --- | --- |
|  |  |  |  |  |  |  |  |  |  |  | **Color Temperature** | **Electrical Power** | **Green/Red-Free Mode** |
| *Make*  *Model* | *Units* | *kilograms* | *X* | *line pairs per mm* | *mm* | *mm* | *mm* | *Mechanism Type* | | *hours per charge* | *Kelvin* | *Watts* | *type* |
| Leisegang  Optik 2 | | 28.5^X^ | 3.75  7.5  15 | 14  20  29 | 76  38  19 | 22  23  23 | 300 | Manual | Stand | N/A | 5000-5500 | 10 | Flip-Down Barrier Filter |
| MobileODT’s  EVA | | 0.508^*^ | 3.8  4.0 | 12  12 | 54  106 | 34  17 | 450  250 | Manual | Handheld or Stand | 10 | 6500 | 3 | Electronic Green Channel |
| Gynius AB’s  Gynocular | | 0.480^*^ | 5  8  12 | 25  40  60 | 40  30  20 | 9  5  2.5 | 300 | Manual | Handheld or Stand | 4 | 2700-3000 | N/A | Green LED |
| Pocket Colposcope Generation 3 | | 0.271* | 3^+^  30 | 10  72 | 55  7 | 12.5  1 | 50  5 | Auto & Manual | Handheld | 12 | 5500 | 2 | Green LEDs |
| Pocket Colposcope  Generation 4 | | 0.109* | 3^+^  30 | 10  72 | 55  7 | 12.5  1 | 50  5 | Auto & Manual | Handheld | 8 | 5500 | 0.5 | Electronic Green Channel |

A superscript “x” indicates that value listed is the manufacturer reported weight for colposcope and stand, but doesn’t include computer tower nor flat screen monitor. A superscript “*” indicates that the value listed is the manufacturer reported weight for colposcope, but doesn’t include laptop or smartphone. A superscript “+” indicates that the range of values listed are for a continuous range of optical magnifications. All other colposcopes have only a discrete set of magnification values as reported in the table. “N/A” indicates that the values are not available from the manufacturer.
